# Understanding the Perceived Relationship between Sun Exposure and Melanoma in Atlantic Canada: A Consensual Qualitative Study Highlighting a “Sunscreen Paradox”

**DOI:** 10.3390/cancers15194726

**Published:** 2023-09-26

**Authors:** Sauliha Alli, Jonathan LeBeau, Agustina Hasbani, François Lagacé, Ivan V. Litvinov, Sandra Peláez

**Affiliations:** 1 Temerty Faculty of Medicine, University of Toronto, Toronto, ON M5S 1A8, Canada; sauliha.alli@utoronto.ca; 2 Faculty of Medicine and Health Sciences, McGill University, Montreal, QC H3T 1C5, Canada; jonathan.lebeau@mail.mcgill.ca (J.L.); agustina.hasbani@muhc.mcgill.ca (A.H.); francois.lagace@mail.mcgill.ca (F.L.); 3 School of Kinesiology and Physical Activity Sciences, Faculty of Medicine, University of Montreal, Montreal, QC H4A 3J1, Canada; 4 Research Centre of Sainte-Justine University Hospital, Montreal, QC H3T 1C5, Canada

**Keywords:** melanoma, skin cancer, behavior, risk factors, sun exposure, sun protection, skin cancer prevention, Atlantic Canada, sunscreen paradox

## Abstract

**Simple Summary:**

Melanoma is a form of skin cancer that can be prevented by limiting exposure to the sun. Among the Atlantic provinces of Canada, varying incidence rates of melanoma have been reported. The province of New Brunswick has melanoma incidence rates comparable to the national average, while in Nova Scotia and Prince Edward Island, the rates are above the national average, and in Newfoundland and Labrador, the rates are below. We conducted focus groups with participants in Atlantic Canada to understand the factors contributing to this difference. We found that Canadians living in Nova Scotia and Prince Edward Island—provinces with high melanoma incidence rates—were more likely to report using sun protection, more aware of the health risks of sun exposure, and more apt to follow the UV index. Despite this, they also received more sun exposure due to warmer temperatures and a tendency to engage in outdoor occupations and recreational activities. These findings suggest a sunscreen paradox, whereby individuals with higher levels of sun exposure also tend to use more but not an adequate quantity of sunscreen or other sun-protection measures, providing a false sense of security. Tailored sun protection campaigns must consider this sunscreen paradox and the unique norms of communities in Atlantic Canada and elsewhere in the world to design effective messaging.

**Abstract:**

Globally, cutaneous melanoma (CM) incidence is increasing, with sun exposure identified as a key modifiable risk factor. The Atlantic provinces of Canada display varied CM incidence rates: New Brunswick aligns with the national average, while Nova Scotia and Prince Edward Island exceed it, and Newfoundland and Labrador fall below this benchmark. We investigated the relationship between sun exposure and CM in these provinces. Twenty-two focus groups encompassing 95 Atlantic Canada residents were conducted and transcribed. A thematic analysis was conducted in MAXQDA using the social–ecological model as a framework. Residents of high-CM incidence provinces demonstrated greater sun exposure awareness, consulting UV indices, and using sunscreen and sun-protective clothing. However, they received greater UV exposure due to warmer climates and outdoor work and cultural activities. Conversely, those in low-incidence provinces used sunscreen and sun-protective clothing less often, engaged in occupations and hobbies affording less sun exposure, and lived in cooler climates. Our data supports a possible “sunscreen paradox”, whereby increased sunscreen use is correlated with augmented sun exposure, leading to a deceptive sense of security. Public health initiatives in Atlantic Canada promoting sun safety must address this paradox while integrating community-specific behaviors and norms in order to develop tailored campaigns.

## 1. Introduction

Cutaneous melanoma (CM) is one of the most aggressive forms of skin cancer, with a propensity for metastasis and poor prognosis [1,2]. The estimated economic cost of skin cancer in 2004 was CAD 532 million, with melanoma comprising 83.4% of this cost [3]. Its incidence rate has been reported to be on the rise in Canada and around the world [4,5,6,7,8,9]. Recently, we identified differences in CM incidence across Canadian provinces, with Nova Scotia (NS) (incidence rate of 27.66 cases per 100,000 individuals per year) and Prince Edward Island (PEI) (30.94) having age-standardized incidence rates of CM above the national average (20.75); New Brunswick (NB) (19.99) having incidence rate similar to the Canadian average; and Newfoundland and Labrador (NL) positioning below the national average (16.63) [7].

Within the Canadian population, several sociodemographic variables have been associated with sun exposure rates and subsequent CM development, including geographic latitude, climate, vegetation, genetic predisposition, Fitzpatrick skin phototype, socioeconomic status, occupation, and behavioral habits [7,10,11,12]. Indeed, extensive evidence reveals that CM is largely preventable with the use of sun protection measures [13,14,15], such as the use of sunscreen and sun-protective clothing as well as avoidance of intense sun, sun tanning and sunburns. Nevertheless, with regard to the former, a paradox has emerged in the sun protection literature that those who use sunscreen do not have lower odds of sunburn, despite taking notice of sun protection and skin cancer prevention messages [16,17,18,19,20].

Given the geographic proximity and the sense of regional identity that has been well documented [21,22,23], it is appropriate to consider the Atlantic Region as an important geographic and cultural unit in Canada. Thus, investigating sociocultural aspects as per people’s perspectives regarding sun exposure may contribute to a better understanding of the collected evidence in recent quantitative studies [8,11,12]. This may ultimately help shape public health campaigns that support sustained behavioral change.

### 1.1. Theoretical Framework

The social–ecological model—developed by Bronfenbrenner in the 1970s [24]—has been widely used in public health and social health research. The Centers for Disease Control and Prevention has adapted it as a framework to understand and guide initiatives related to disease prevention [25]. The model states that behaviors are influenced by the interaction between different systems at individual, interpersonal, community, and societal levels [24]. The microsystem consists of direct and informal interactions and relationships and is thought to be the most influential at the individual level. The mesosystem refers to formal interactions, such as those that take place at school, work, and in the neighborhood. The exosystem includes both formal and informal indirect influences that affect a person even when he or she has no direct relationship with the entity (e.g., the implementation of sunscreen price policies to enhance its use). The macrosystem is the sociocultural environment in which a person lives and the values that are included. Lastly, the chronosystem consists of time, historical context, and policies [26]. This model could be used as a framework for understanding CM risk in Atlantic Canada.

### 1.2. The Current Study

Up to now, large-scale qualitative studies have not examined behavioral drivers behind sun exposure and skin cancer awareness in Atlantic Canada. In this context, the objective of this study was to explore the individual, interpersonal, community, and societal factors influencing sun exposure habits perceived by individuals living in the provinces of Atlantic Canada. Using a consensual qualitative research design, we aimed to understand participants’ perspectives of sun exposure to serve as a foundation for the development of public health recommendations for CM prevention.

## 2. Methods

### 2.1. Study Design

The present study is part of a research program (SunFit Project) designed to: (a) explore adults’ perspectives regarding sun protection habits and CM and (b) develop tailored sun exposure recommendations to enhance behavior change regarding sun exposure practices leading to the prevention of CM. Using a patient and public involvement strategy, we designed a consensual qualitative research study [27]. This type of study uses an inductive method characterized by open-ended data collection in small samples in which the importance of context, the integration of multiple viewpoints, and the consensus of the involved parts are at the core of the design. This design is especially well-suited for research that requires rich descriptions of inner experiences, attitudes, and values. Participants including CM patients and family members, were invited to join the research team at the stage of this proposal’s inception. While the project was evolving, the specific needs of knowledge users were addressed upon request and discussion. The entire team participated in the design and implementation.

### 2.2. Participants and Procedures

The SunFit cohort [11], which includes a total of ~7500 individuals, was used to distribute an informational newsletter, in which the SunFit qualitative study was described and contact details were provided. Two hundred and fifty adults replied, indicating their interest in participating. Further recruitment was carried out using word of mouth and the snowballing technique [28]. People interested in participating contacted the research team by email. They were asked to complete a form reporting personal (i.e., name, date of birth, postal code, and contact) details and demographic information (optional), as well as their preference for participating in the study either virtually or in person. Participants were included in the study if they were: ≥16 years of age, lived in one of the four Canadian Atlantic provinces (NS, PEI, NB, or NL), and were able to speak either English or French. In total, 95 participants attended the proposed focus groups.

The McGill University Faculty of Medicine and Health Sciences Institutional Review Board provided ethical approval in May 2022 (Protocol # A05-B39-22A). Participants were offered monetary compensation of CAD 10–50 for their participation in this study. The data collected and the participants’ identities were securely saved in SP’s University-based iCloud.

### 2.3. Data Collection

One virtual and 22 in-person focus groups were conducted between July and August 2022. We used this data collection technique because we were interested in open discussion and exchange of ideas among participants [29]. The summer months were selected because this is the time of the year when people are more aware of sun protection habits [30]. In the case of the present study, two medical students and a college student (two females and one male: SA, JL, and AH, respectively), trained in qualitative research, acted as moderators, participant observers, and note-takers. Participants were prompted to discuss their sun-protection behaviors in the context of sun exposure. We assumed an iterative, evolutive, and consensual approach, meaning that the original topic guide evolved based on observations, field notes, and debriefing among team members during data collection.

Prior to starting the focus groups, participants were asked to sign either an online or in-person informed consent form detailing the risks and benefits of participation and were given an opportunity to ask questions about this study.

### 2.4. Data Analysis

The discussions that took place during the focus groups were recorded and ten verbatim transcribed by AH and uploaded into MAXQDA software Version 2022 [31]. The transcriptions were verified against the audio by SP [32]. The three research students read the transcripts thoroughly to become familiar with the discussions held. A thematic data analysis was conducted [32]. Codes (i.e., small meaningful segments) were identified, grouped together based on identified patterns, contrasted, and compared. Codes were grouped into themes (i.e., abstracted data representing the main outcome of analysis), inspired by the previous literature, ideas discussed by the participants, and researchers’ reflections. The research team met via Zoom once per week to share their reflections on data analysis, discuss the evolving coding system, and contribute to the analysis being conducted. Every two weeks, preliminary results were shared with the rest of the research team. This strategy helped to enhance the process of reflection.

## 3. Results

### 3.1. Participants’ Characteristics

A total of 13 focus groups from NS and 2 from PEI were included in the high-CM-incidence group consisting of 74 participants; 5 focus groups from NB were included in the average-incidence group consisting of 11 participants; and 2 focus groups from NL were included in the low-incidence group (6 participants). The mean age of participants was 65.0 in the low-incidence group, 70.0 in the average-incidence group, and 69.4 years in the high-incidence group. Demographic data on the participants are summarized in Table 1.

### 3.2. Participants’ Perspectives Regarding Sun Exposure, Sun Protection, and Melanoma

We analyzed reported behaviors regarding sun exposure, sun protection, and melanoma as perceived by participants and per the relationship to their social environment. More specifically, critical environmental factors discussed by participants, such as dominant industries and occupations (the mesosystem), recreational activities and regional traditions (the exosystem), and climate and geographic conditions (the macrosystem) were related to sun protection habits enacted and reported by individuals (the microsystem) (Table 2).

## 4. Sun-Protection Habits

### 4.1. Sunscreen

Across all groups, there was a range of behaviors from diligent and proactive sun protection to a more reactive approach based on expected sun exposure. However, the NS and PEI groups showed more regular habits of sunscreen use, possibly due to a higher awareness or due to environmental factors [12]. For example, a participant from PEI said:

“I always have sunscreen in my purse in case I get caught out. You know, they were samples that were given to me, but I keep them here in case I end up at the beach unexpectedly and need to slap something on, right?”.

(Focus Group 3, PEI)

Individuals from NB, on the other hand, seemed to be more proactive and consistent in their habits, often applying sun protection even for shorter outdoor activities. They also seem to show a slightly higher awareness of the UV index and its implications for sun protection. One participant shared, “Sometimes I even use SPF 60 because I burn so easily” (Focus Group 20, Saint John, NB).

Meanwhile, individuals from NL displayed a more activity-dependent approach to sun protection, sometimes not considering less obviously risky activities as requiring sun protection.

### 4.2. Sun-Protective Clothing

In addition to sunscreen, participants used sunglasses, sun-protective clothing, hats, and umbrellas. Sunglasses use was not discussed in any of the NL groups, but both the NS/PEI and NB groups demonstrated an understanding of the importance of wearing sunglasses for sun protection. However, the emphasis was slightly different in each group: NB participants seemed to stress more the quality and type of sunglasses in relation to their benefits for sun protection, while those from NS and PEI mentioned sunglasses usage more in relation to specific outdoor activities and personal health concerns. In this regard, a participant said: “And the other important thing is to wear the right sunglasses that will protect your eyes. You don’t want to buy these cheap colored glasses you need proper Polaroid lenses” (Focus Group 18, Saint John, NB).

With respect to sun-protective clothing and hats, in NL, there was a general aversion to wearing hats, especially during the summer months, due to personal discomfort with having something on the head. Long-sleeved clothing was preferred instead. NS/PEI and NB, on the other hand, had a more consistent habit of using hats for sun protection, including wide-brimmed hats, baseball caps, and Tilly-like hats, regardless of the season or activity. They also endorsed wearing long-sleeved clothing. For instance, while discussing the use of a hat, a participant said: “No …I just don’t like anything on top of my head. I’ll wear it—sorry—in the wintertime if I’m going for a walk for warmth. But definitely not in the summertime” (Focus Group 17, St. John’s, NL).

Umbrellas were not discussed extensively among participants and were mentioned only in NS/PEI as a means of sun protection.

### 4.3. Shade

Seeking shade was another form of sun protection described by participants. All three groups emphasized the importance of avoiding peak sun hours, typically between 10 AM and 2 to 3 PM, and scheduling outdoor activities outside of these times. However, the motivation for avoidance varied between groups. In NL, avoiding intense sun was motivated by not having to use sunscreen. With regards to this, one participant commented:

“I’m not 100% about anything. I guess the thing that I’m most conscious of all the time, is what time of day it is. Because I know you don’t need to have sunscreen at 9:00 AM morning. You know, just the average times”.

(Focus Group 17, St. John’s, NL)

In NB and NS/PEI, however, avoiding heat was cited as a motivating factor for avoiding sun exposure. A participant noted: “I never look at the UV index but there are days I remember going outside and just feeling literally assaulted by the sun. It’s just so intense” (Focus Group 08, Halifax, NS).

All groups shared an awareness and proactive behavior of seeking shade as a means of sun protection. In NS/PEI and NB, this was connected to a balance between enjoying the outdoors and safety and the recognition that seeking shade was related to a reduced risk of cancer. A participant noted:

“I usually seek the shade myself, so if we do go to the beach or something like that, I try to pick up a shady spot, or I have a chair with an umbrella. I am even a little more cognizant of it to having gone through colon cancer, so I mean, funny how it takes something like that to wake you up and shake you up”.

(Focus Group 08, Halifax, NS)

### 4.4. Avoidance of Sun Tanning and Experience of Sunburn

All groups seemed to show an intentional avoidance of sun tanning. However, in NS/PEI and NB, this was more commonly achieved with active avoidance, whereas in NL, participants did not seem to seek out sun tanning in the first place. A participant noted: “I just...I’ve always probably been fairly careful about the sun just I would never be sunbathing or anything like that” (Focus Group 18, Saint John, NB).

Of note, while participants from all provinces shared past experiences including “sunburns”, the discussion around this was more extensive among people living in NB and NS/PEI, who described blistering, peeling skin, and feeling horribly sick. Sunburns in these groups were significant enough to discourage subsequent sun exposure, as stated in the following quote:

“I remember as a teenager I love[d] the sun and I would go to the beach with my friends and put on sunscreen. One time I fell asleep in the sun, and I woke up and I had such a bad sunburn on my face, it blistered the next day and that was sort of the turning point for me”.

(Focus Group 10, Halifax, NS)

## 5. Dominant Industries and Occupations

Participants from all provinces expressed that the Atlantic provinces, compared with the rest of Canada, have a higher proportion of rural population and economies heavily reliant on natural resources, which often involves outdoor work. Fishing, farming, and construction were mentioned in all four provinces. In the low-incidence province (NL), however, fishing and construction appeared to be the dominant industries. In the average-incidence province (NB), there was a focus on occupations related to forestry including lumber and dockyards. In contrast, in NS and PEI, there was a focus on farming and commercial fishing, including lobster fishing.

Working on the water, which was more common in the high-incidence provinces, was viewed as particularly risky due to the absence of shade and enhanced sunlight refraction in open water. In high-incidence provinces, the concept of a “farmer’s tan” was frequently mentioned and was linked to the perception that farmers and fishermen often neglect sun exposure.

One participant from St. John’s, NL, recounted an observation of outdoor workers, highlighting the intense exposure they endure:

“And there were all these construction workers outside and I was thinking man I should have like, got some water, cold, and just dropped it off. Cause they were looking so, so hot…And especially you think about, growing up, the fishermen out on the water. They often too, like their faces, and their arms, their neck, their heads were always very burnt or tanned”.

(Focus Group 16, St. John’s, NL)

## 6. Recreational Activities and Regional Traditions

As a result of the short summer days across Atlantic Canada, participants from all provinces felt a collective urge to make the most of sunny days and spend time outdoors. One participant from NL noted, “You can’t stay inside on a nice day, because you’ve been inside all winter”. (Focus Group 16, St. John’s, NL). Yet another participant from NB said, “We have a very short summer here in New Brunswick, so we have to take advantage of it” (Focus Group 18, Saint John, NB). Finally, a participant from NS said, “I would rather be outside than in. The winters are long in Canada. We wanna be outside … we also wanna be safe” (Focus Group 11, New Glasgow, NS).

Outdoor hobbies on the water and land were common in all groups, including boating, swimming, fishing, gardening, hiking, biking, golfing, visiting the beach, and attending festivals. Participants from NL described berry-picking and attending the St. John’s Regatta (a rowing event and festival) as common outdoor hobbies. Cross-country skiing was also a popular winter sport specifically in NL and NB. In NB, there was also an emphasis on land activities, including soccer and baseball, lawn bowling, four-wheeling, and hunting. A participant shared, “New Brunswick is one of the most rural provinces in Canada and its forestry is the biggest industry. So, spending time in the woods might be higher than in other provinces” (Focus Group 20, Saint John, NB).

Participants from high-incidence provinces seemed to perceive themselves as “outdoor people”. In fact, one participant even described feelings of guilt for not intentionally spending time outside: “And, and I think we’re a culture of people that like to be outside, you know, we enjoy, you know, boating and gardening and everything outside” (Focus Group 06, Halifax, NS). Also, another participant added: “I’m guilty of that, so cycling and hiking and playing outdoors in the summer, there will be times when I’ll be an hour or two in the sun” (Focus Group 08, Halifax, NS).

## 7. Climate and Geographic Conditions

### 7.1. Temperature

Participants from all groups unanimously agreed that summers in Atlantic Canada were shorter compared with the rest of the country. However, participants perceived the climate in NL and NB as being cooler, with lower temperatures and shorter summers than NS and PEI. Based on these climatic conditions, they perceived their risk of skin cancer to be lower. One participant commented:

“When growing up, I always thought that [in] NL we wouldn’t get skin cancer because we had so little sun. I think we have the least amount of sun of any province per year. With the fog, and it rains a lot”.

(Focus Group 16, St. John’s, NL)

There was also a tendency among both Newfoundlanders and New Brunswickers to consider their weather as being unpredictable, which reduced awareness of sun exposure risk. With regard to this idea, a participant commented, “The environment here is not certain, you don’t think of people getting sunburn. Because of the fog and you know …” and another added, “We are more likely to need a sweater at our beach than sunscreen” (Focus Group 20, Saint John, NB).

### 7.2. Rain

There also seemed to be a difference in the perceived number of rainy days across Atlantic provinces [12]. The phrase “rain, drizzle, and fog” was frequently used by Newfoundlanders to describe their weather on a typical day and by New Brunswickers, albeit less often. While participants in NS and PEI also described their weather as rainy, they did seem to perceive rain as a characteristic feature of their weather. A participant from NS shared, “I moved in [here] 1985. I used to joke what follows 2 days of rain in NS… Monday” (Focus Group 07, Halifax, NS). Another participant from NL described their weather as, “Rain, drizzle and fog. Especially here, especially here” (Focus Group 16, St. John’s, NL).

### 7.3. Temperature and UV Index

Temperature and the UV index were discussed in the groups. Participants from NL identified that their winters are not as severe as they used to be. In NS, participants noted that temperatures remained elevated for longer hours during the day. Interestingly, there seemed to be greater consciousness about increasing UV levels among participants from the high-incidence groups and the consequent sun exposure risk this conferred. Participants from these groups also reported checking the UV index more often compared with the low-incidence groups, in which this was not mentioned at all. As an example, one participant from NS commented, “Come May/June I start to look at UV indexes and listen to them on the on the radio” (Focus Group 08, Halifax, NS).

In the average-incidence group, there was also mention of checking UV indexes and an awareness that the UV index tended to be high in NB despite the fog, which was thought to contribute to sunburn or skin cancer. With respect to this idea, one participant noted, “Another thing people have to be aware of is that the UV comes through the fog. We have a lot of fog here. So, you cannot say because it is foggy you are not getting exposed” (Focus Group 18, Saint John, NB).

### 7.4. Climate Change and Ozone Depletion

Global warming/climate change emerged as a theme in conversations in all groups. In NB and NS/PEI, participants reported a rise in temperatures for longer durations over the years. One participant said: “We didn’t even ever get the days that we are getting now like over 30 or 35 for a couple of weeks. That didn’t happen it would normally be like a week tops” (Focus Group 19, Saint John, NB).

However, participants from NL seemed to perceive that NL would be protected from the effects of global warming/climate change. One participant from St. John’s mentioned that while most regions were expected to see a rise in temperatures, NL was expected to see a decrease:

“And a couple of years ago, I’m looking over to the side… People think I’m making it up. When they were talking about global warming and how the whole earth is becoming so warm, there’s one area in the whole world that’s going to be colder and is right around NL”.

(Focus Group 16, St John’s, NL)

The depletion of the ozone layer over time, and the consequent increase in exposure to UV radiation, was also an idea that emerged in NL and NS/PEI. However, in NL, the discussion centered around the idea that the ozone layer was not a tremendous priority, and possibly an issue of the past, while in PEI and NS focus groups, in that context, it was also thought to be an ongoing and urgent issue. Accordingly, one participant from NL commented:

“I haven’t thought about the ozone in forever, because it’s just not talked about in the same way, but when there was all this worry about the depleting ozone, it was like we’re all gonna end up with skin cancer because we don’t have that built-in exposure” barrier.

(Focus Group 17, St. John’s, NL)

In contrast, a participant from NS said:

“Over the course of my life I’ve got some nasty sunburns, but we didn’t think of the sun as being something so dangerous, and you didn’t get sunburns as quickly as we do now because it wasn’t as much of a problem with the ozone layer”.

(Focus Group 11, New Glasgow, NS)

Changes in the ozone layer did not emerge in discussions in NB.

### 7.5. Rural and Urban Settings

In the high-CM-incidence focus groups, as highlighted above, the idea emerged that Atlantic Canada had a much larger rural population than the rest of the country. Participants felt that the economies in rural areas tended to rely more on natural resources and would in turn have more outdoor work and activities, where people were less likely to use sun protection and have less awareness of the risks of sun exposure. One participant from NS shared:

“One factor for Atlantic Canada … is that we have a much larger rural population than Canada as a whole… People in rural settings tend to be natural resource economy to a large extent. So farming, fishing, lumbering, what have you, but basically outdoor activity and physical work activity … so there’s likely more exposure and to some degree, probably less awareness as well”.

(Focus Group 10, Halifax, NS)

In the average-incidence group, there was also the idea that NB’s rural settings confer more sun exposure. A participant said, “As a kid in Saint John and rural wise like you would get hot, you’d be laying on the beach in it would get hot and you want [to] cool [down]” (Focus Group 10, Saint John, NB). In the low-CM-incidence focus groups, there were no discussions around rural versus urban settings or the implications of sun exposure.

### 7.6. Public Expectations for Intervention

In all provinces, participants reported an interest in a public health campaign tackling skin cancer risks. When asked to suggest potential approaches to reduce CM incidence, participants described interventions at the individual, interpersonal, community, and societal levels (the microsystem, mesosystem, exosystem, and macrosystem, respectively), which are summarized in Table 3 below. Government and policy level changes and encouraging indoor activity were more frequently cited in high-CM-incidence groups, whereas the low- and average-CM-incidence groups described more community and interpersonal access approaches including festivals, community organizations, education, UV stickers (a sticker worn on the body providing quantitative feedback on sun exposure levels) and removing access barriers to sun-protection methods.

When asked to provide examples of the content of messaging, participants from all groups discussed beauty, healthy living, weather, and global warming. Participants in the higher incidence groups opted for more scary, graphic approaches focused on negative health outcomes, including pictures of skin cancer and stories of cancer survivors. With respect to this idea, a participant from PEI said, “I wished I knew more. I was so happy when I do see pictures of tumors just so I know what to look for in my own body. But you know, that sort of education” (Focus Group 03, PEI).

## 8. Discussion

### 8.1. Summary of Results

There has been a growing interest in understanding the knowledge, attitudes, and perceptions that influence sun exposure at both individual and systemic levels, which is a relationship that has not been extensively studied in Atlantic Canada. According to the social–ecological model (SEM), health behaviors are influenced by the interactions at the microsystem (individual), mesosystem (interpersonal), exosystem (community), and macrosystem (societal) levels [24]. In accordance with this theoretical framework, we identified several factors that influenced sun exposure in the Atlantic provinces of Canada. At the individual level, our study revealed that Canadians residing in high- and average-CM-incidence provinces (NS/PEI) use sunscreen and sun-protective clothing (e.g., sunglasses, UV protective clothing), have more of a sun tanning culture, and are more aware of the health risks of sun exposure compared with those in low-incidence provinces (NL).

At the interpersonal level, high-incidence provinces had dominant industries involving outdoor work including farming and fishing, and working on the water was also perceived to involve higher sun exposure risk. At the community level, outdoor hobbies, although popular in all Atlantic provinces, appeared to be more integral to the self-identity of NS/PEI residents, which may suggest the presence of cultural forces to increase sun exposure. Finally, at the societal level, the high-incidence provinces experienced more favorable climate conditions, with more sunny days, higher temperatures, and fewer rain events, and had more rural geographies. Taken together, these findings suggest that while higher-incidence provinces have a greater awareness of the risks of sun exposure, and use more sun protection, they paradoxically experience greater levels of sun exposure.

### 8.2. Microsystem: Sun Protection Habits

Our previous research demonstrated a higher incidence of skin cancer in NS and PEI compared with the rest of Canada and NB and a lower incidence in NL [33]. This study noted that individuals from regions with higher CM incidence tended to use sunscreen and sun-protective clothing more commonly than individuals from low-CM-incidence regions but were similar in their tendency to seek shade. Interestingly, quantitative studies have shown that the use of sun-protective clothing and avoidance of the sun may confer greater photoprotection than sunscreen [34,35,36]. The preference for wearing sunscreen in high- versus low-CM-incidence provinces may contribute to lower overall sun protection received. In addition, a more prominent sun tanning and beach culture seemed to be present in NS/PEI, and participants from this group, along with those from NB, described more experiences with sunburns. This is consistent with the findings of a large US-based prospective cohort study (*n* = 75,614), in which 13.1% of beachgoers reported sunburn, with the highest sunburn rates occurring at beaches along the Eastern Seaboard [37], an area that is geographically proximate to Atlantic Canada.

The curious finding that high-CM-incidence provinces have high sunburn rates despite high sunscreen use rates resonates with the existing sun protection literature. One Norwegian study reported that sunscreen users experienced significantly more sunburns, took more sunbathing vacations, and were more likely to use indoor tanning devices [13]. In a Swiss study of elementary school children, regular sunscreen use and higher sun-related knowledge surprisingly predicted sunburn [38]. These findings are consistent with the idea of a “sunscreen paradox”, whereby those who are more aware of the risks and take protective measures also expose themselves more frequently to sunlight [16,17,18,19]. Therefore, while sunscreen use may protect against direct UV damage, it may inadvertently encourage prolonged sun exposure due to a false sense of security. This could partly explain the higher incidence of skin cancer in areas where sunscreen use is more prevalent.

### 8.3. Mesosystem: Dominant Industries and Occupations

Across all Atlantic provinces, there was a predominance of outdoor occupations. It is well established in the literature that professionals who are employed in outdoor occupations are exposed to higher levels of UV radiation [39] than those who work indoors. Occupational UV exposure has specifically been correlated with the development of cutaneous squamous cell carcinoma [40] and less commonly, basal cell carcinoma [41]. In fact, the International Commission on Non-Ionizing Radiation Exposure (ICNIRP) statement on exposure of workers against UV exposure identified a study where the level of UV exposure, measured in terms of standard erythemal dose (SED), the amount of UV required to produce mild erythema to the skin, was compared for indoor and outdoor workers. Outdoor workers in Northern Europe were found to receive about 400–600 SED of UV exposure per year, which was two to three times the exposure dose of indoor workers [39]. The findings were corroborated by Wittlich and colleagues in 2016, who estimated an annual occupational sun exposure of 538 SED annually, and a lifetime exposure of 8417 SED, for outdoor workers [40].

However, participants from NL often listed construction as an occupation, and those from NB listed construction and forestry, whereas NS/PEI listed farming and occupations on the water, including commercial and lobster fishing. Daily levels of UV exposure have accordingly been observed to be high in farmers [41,42], with measures of approximately 14.5 SED in Italy [43], and slightly lower in construction workers (9.9 SED in Australia) and fishermen (up to 5.4 SED in Italy) [44]. Consistent with these findings, 2016 data from Statistics Canada revealed that in terms of the subsectors contributing to nominal GDP, hunting, fishing, and water made up the greatest proportion of the NS/PEI economy, whereas for NB, it was mining and forestry, and for NL, it was crude oil [45]. These differences in occupational sun exposure, as discussed by focus group participants, may in part explain patterns of high vs. average vs. low incidence of skin cancer across Atlantic Canada.

### 8.4. Exosystem: Recreational Activities and Regional Traditions

With respect to recreational activities, participants from NS/PEI more often discussed visiting beaches as a pastime, which represents a form of recreational sun exposure. In fact, in our recent work using quantitative survey data, recreational sun exposure was more frequently reported in PEI and NS [11]. Several studies have also shown that the refraction of sunlight is higher for water and sand compared with grass, which supports higher sun exposure levels at beaches [39,46]. Interestingly, the perception of being an “outdoor person”, as reported by our participants from NS/PEI, may point to cultural norms encouraging more sun exposure. This, combined with feelings of guilt for not being outdoors/exposed to the sun may indicate a closer connection between sun exposure and self-perception.

### 8.5. Macrosystem: Climate and Geographic Conditions

The influence of climate on sun exposure behaviors and skin cancer incidence, as observed in our study, is supported by the existing literature. Globally, there is a recognized inverse relationship between CM incidence and geographic latitude, with areas further from the equator having lower incidence rates of melanoma [47]. Correspondingly, St. John’s, NL, situated at a higher latitude and thus further from the equator than NB, NS, and PEI, experiences lower average summer temperatures (8–16 °C) compared with NS/PEI (14–18 °C) and NB (14–16 °C) [48]. This concept was underscored continuously in our focus groups. Consistent with findings from Australia, these increased temperatures may be associated with individuals spending more time outdoors and experiencing a higher incidence of sunburns [49].

We recently identified that higher general annual temperature (i.e., more pleasant ambient temperature), availability of green spaces, and the UVR index are the three main factors associated with CM risk in Canada [12]. In 2018, lower average greenness and average normalized vegetation index were reported in NL (92.2 and 0.67, respectively), compared with NB (94.7 and 0.71, respectively), NS (93.8 and 0.72, respectively), and PEI (94.0 and 0.68, respectively) [50]. Our qualitative data parallels these findings, with more participants from high- and average-incidence provinces discussing experiences in rural settings and reporting checking the UV index. They also more frequently described concerns about climate change and ozone depletion, which may suggest a greater understanding of the risk of sun exposure.

### 8.6. Strengths and Limitations

We used a consensual qualitative research study design to understand the factors contributing to high and low incidence of cutaneous melanoma within Atlantic Canada. To the best of our knowledge, this is the first study to analyze the experiences of communities in Atlantic Canada, which has an especially high burden of skin cancer. Our focus groups were conducted on the older population within Atlantic Canada who may have a higher risk of CM and also reflect the broader demographic of the Atlantic provinces. The analysis was conducted using a carefully constructed theoretical framework while maintaining openness and flexibility in interpreting the outcomes. One notable limitation of this study was the size of our sample, which consisted of 22 focus groups that largely represented participants from high-incidence provinces (15 interviews) with considerably less representation from the low-incidence province of NL (2 interviews). While this allowed us to better study the enacted behaviors of the high-CM-incidence group, it may have limited our ability to understand attitudes and behaviors in NL. We also used a snowball sampling strategy, which may have led to an overinclusion of individuals within the same networks or communities. Such homogeneity could lead to shared perspectives, thereby potentially narrowing the diversity of experiences and views captured. Consequently, these biases could impact the broader generalizability of our findings. We also acknowledge that the efficacy of sunscreen in real-world applications may be compromised if not applied appropriately, leading to suboptimal protection against sunburn. This potential inconsistency in the application was not explored in this study but might contribute to the observed sunscreen paradox.

### 8.7. Implications for Education, Clinical Practice, and Research

Individual behaviors are crucial determinants of solar UV exposure, including actions such as wearing protective clothing, sunglasses, and hats, utilizing sunscreen and seeking shade. Our findings underscore the need for comprehensive public education regarding the risks associated with sun exposure, the specific dangers posed by occupational and recreational exposures, and the appropriate use of sunscreen.

The participants in this study demonstrated awareness of preventive measures for skin cancers. Interestingly, community members from the high-incidence group (NS and PEI) cited more societal-level factors contributing to CM risk and were interested in scarier messages focused on possible negative health outcomes. This is consistent with their greater awareness of skin cancer and their use of sun protection. Perhaps this higher awareness translates into the perception that skin cancer is a broader issue that requires system-level intervention and the participation of governments.

Public health campaigns need to be carefully crafted, considering the unique cultural norms prevalent in areas of high-, average-, and low-CM-incidence regions. For the high- and average-incidence groups, it may be valuable to focus on a reduction in recreational sun exposure with the use of sun-protective clothing as opposed to mostly relying on sunscreen, which perpetuates the “sunscreen paradox”. Sun-protective UPF > 50 clothing has shown greater efficacy in preventing sunburns and providing reliable sun exposure [36]. Based on our findings and interviews, efforts to limit outdoor activities in NS/PEI may not receive support based on community norms.

For the low-incidence group, it may be valuable to center efforts on educating the public on UV exposure risks in the absence of direct sunlight. Beyond individual behaviors, policy changes are needed to encourage the provision of public shade through government subsidies, employer-sponsored sunscreen/clothing and personal protective equipment, and removing tax from sun-protective items, making them more affordable to the public and particularly for outdoor workers [10]. The development of skin cancer prevention and screening guidelines could serve as an essential measure in primary care. Given the limited research conducted in Atlantic Canada, further investigation is needed to understand the distinctive behavioral-, community-, and system-level challenges that contribute to high melanoma incidence rates in this region.

## 9. Conclusions

In summary, our study found that the high incidence rate of CM observed in NS and PEI compared with NB (rates similar to the national average) and NL (rate below the national average) may be explained by four factors: (1) greater use of sunscreen, which confers a false sense of security, known as “the sunscreen paradox”, (2) a predominance of outdoor occupations involving higher sun-exposure levels, (3) a culture of engaging in outdoor hobbies/activities, and (4) warmer temperatures increasing UV exposure. Effective behavioral change interventions to address these gaps should be tailored to the unique social and cultural experiences of each group while also addressing systemic barriers that create broader access challenges across Atlantic Canada.

## Figures and Tables

**Table 1:** Focus group participant demographics from NL, NB, NS, and PEI.

Demographic Factor	N
Age (Mean ± SD)	67 ± 8
Gender	
Woman (N (%))	38 (71.7)
Man (N (%))	15 (28.3)
Ethnicity (N (%))	
White Non-Hispanic or Euro-Canadian	51 (96.2)
Black, Afro-Caribbean, or African	1 (1.9)
Other	1 (1.9)
Household income after taxes (N [%])	
<CAD 20,000	2 (3.8)
CAD 20,000–49,999	7 (13.2)
CAD 50,000–69,999	10 (18.9)
CAD 70,000–89,999	9 (17.0)
≥CAD 90,000	17 (32.1)
Declined to answer	8 (15.1)
Highest level of education (N (%))	
High school complete	8 (15.1)
College or CEGEP	13 (24.5)
Undergraduate	20 (37.7)
Graduate or higher	12 (22.6)
Personal history of skin cancer	9 (17.0)
Family history of skin cancer	25 (48.1)

**Table 2:** Key factors related to sun exposure in high-, average-, and low-incidence provinces at the microsystem, mesosystem, exosystem, and macrosystem levels.

Key Factor	Low Incidence Province (NL)	Average Incidence Province (NB)	High Incidence Provinces (PEI/NS)
**Microsystem** Sun protection habits	Sunscreen use may be activity-dependent. Little use of sun-protective clothing. Avoid intense sun and seek shade. Low sun tanning culture. No discussion about sunburn.	Sunscreen use routine. Frequent use of sun-protective clothing. Avoid intense sun and seek shade. Possible sun tanning culture. Awareness of health risks of sunburn.	Sunscreen use routine. Frequent use of sun-protective clothing. Avoid intense sun and seek shade. High sun tanning culture. High awareness of health risks of sunburn/tanning.
**Mesosystem** Dominant industries and occupations	Predominately fishing and construction.	Predominately forestry (lumber, dockyards).	Predominately farming and fishing (including lobster fishing).
**Exosystem** Recreational activities and regional traditions	Festivals and winter sports. Incumbent to be outdoors in the summer.	Land activities (soccer, baseball, lawn-bowling, four-wheeling, hunting) and winter sports. Incumbent to be outdoors in the summer.	Beaches, boating, and fishing. A cultural tradition to be outdoors in the summer; self-perception as “outdoor/nature people”.
**Macrosystem** Climate and geographic conditions	Lower temperatures, shorter summers, and fewer sunny days. Climate of rain, drizzle, and fog. Lower awareness about climate change and ozone depletion. Impact of rural vs. urban setting not highlighted.	Lower temperatures, shorter summer, fewer sunny days. Climate of rain, drizzle, and fog. Greater awareness about climate change and ozone depletion. Rural settings connected to greater sun exposure.	Higher temperatures, shorter summers, more sunny days. Climate sunny and rainy. Greater awareness about climate change and ozone depletion. Rural settings connected to much greater sun exposure.

**Table 3:** Approaches endorsed by participants to reduce incidence rates of cutaneous melanoma (CM) in their home provinces at the microsystem, mesosystem, exosystem, and macrosystem levels.

Approach to Reducing CM Incidence Rates	Low incidence Province (NL)	Average Incidence Province (NB)	High Incidence Provinces (PEI/NS)
**Microsystem** UV sticker	Endorsed.	Endorsed with considerable interest.	Endorsed.
**Mesosystem** Education, screening, involvement of doctors’ offices and pharmacies	All methods endorsed.	All methods endorsed. Particular interest in education.	All methods endorsed. Considerable interest in education.
**Exosystem** Sunscreen samples, in-person events (e.g., festivals), community organisations	All methods endorsed.	All methods endorsed.	All methods endorsed. Sunscreen samples and in-person events especially popular.
**Macrosystem** Government and policy change, employers, occupational health, addressing economic barriers	Generally, not endorsed. Interest only in addressing economic barriers.	Generally, not endorsed. Interest only in addressing economic barriers.	All methods endorsed.

## Data Availability

The data presented in this study are available on request from the corresponding author. The data are not publicly available due to privacy and ethical limitations.
